# Host Jumps and Radiation, Not Co‐Divergence Drives Diversification of Obligate Pathogens. A Case Study in Downy Mildews and Asteraceae

**DOI:** 10.1371/journal.pone.0133655

**Published:** 2015-07-31

**Authors:** Young-Joon Choi, Marco Thines

**Affiliations:** 1 Faculty of Biosciences, Institute of Ecology, Evolution and Diversity, Goethe University Frankfurt am Main, D-60438, Frankfurt am Main, Germany; 2 Biodiversity and Climate Research Centre (BiK-F), D-60325, Frankfurt am Main, Germany; 3 Integrative Fungal Research Cluster (IPF), D-60325, Frankfurt am Main, Germany; 4 Senckenberg Gesellschaft für Naturforschung, D-60325, Frankfurt am Main, Germany; Agriculture and Agri-Food Canada, CANADA

## Abstract

Even though the microevolution of plant hosts and pathogens has been intensely studied, knowledge regarding macro-evolutionary patterns is limited. Having the highest species diversity and host-specificity among Oomycetes, downy mildews are a useful a model for investigating long-term host-pathogen coevolution. We show that phylogenies of *Bremia* and Asteraceae are significantly congruent. The accepted hypothesis is that pathogens have diverged contemporarily with their hosts. But maximum clade age estimation and sequence divergence comparison reveal that congruence is not due to long-term coevolution but rather due to host-shift driven speciation (pseudo-cospeciation). This pattern results from parasite radiation in related hosts, long after radiation and speciation of the hosts. As large host shifts free pathogens from hosts with effector triggered immunity subsequent radiation and diversification in related hosts with similar innate immunity may follow, resulting in a pattern mimicking true co-divergence, which is probably limited to the terminal nodes in many pathogen groups.

## Introduction

Two kingdoms, Fungi and Straminipila (mostly Oomycetes), include the vast majority of eukaryotic plant pathogens, of which many, like *Phytophthora infestans* of the oomycetes and *Puccinia tritici* of the rust fungi, are of great economic importance. In both fungi and oomycetes, species richness is high in obligate biotrophic pathogens. To maintain this biotrophic lifestyle, they have developed highly host-specific and sophisticated mechanisms to confront the resistance system of the host plants [1, 2]. As a result, an often strict host specificity, i.e. a given pathogen species can infect and utilize only a narrow range of host plants or only a particular host species, is generally found in most biotrophic species of both fungi and oomycetes. As obligate biotrophic pathogens are mostly highly host specific, and cannot reproduce on dead plant tissue, it could be assumed that host jumps should be rather rare and co-evolution in a strict sense (i.e. co-divergence) should be favoured. However, phylogenetic studies in oomycetes have revealed early that in the largest genera of downy mildews, *Peronospora* [3], *Hyaloperonospora* [4], and *Plasmopara* [5], several host jumps must have occurred to explain the complex phylogenetic patterns observed, considering the high host-specificity for individual pathogen species.

The specialized interactions have been contributing as model systems to elucidate short term co-evolution, as well as genetic and phenotypic diversity of pathogen-plant interactions [6, 7]. In oomycetes, especially *Hyaloperonospora arabidopsidis*, the downy mildew pathogen of the model plant *Arabidopsis thaliana*, and *Bremia lactucae*, arguably the most important pathogen of lettuce (*Lactuca sativa*), are pathogen models useful in elucidating the mechanisms used by a pathogen to establish and sustain a biotrophic relationship with a plant [8–10].

Like the case of other plant pathogens, most studies of obligate pathogen-plant interactions focus on gene-for-gene interactions at the molecular level. However, such investigations are usually limited to the relationships between cultivars or ecotypes of a particular host species and races of a particular pathogen species. Thus, there is still hardly any knowledge of how obligate pathogens have successfully specialized and diversified on the great variety of host plants from a macro-evolutionary viewpoint, although a variety of studies address some co-phylogenetic aspects of fungal pathogens and their hosts [11, 12].

When obligate parasite lineages have been in intimate association with their hosts during their diversification, events that isolate the host populations may also isolate the populations of their associated parasites, and thus speciation in one group is potentially paralleled by speciation in the other [13]. This mode of diversification will result in a pattern of shared evolutionary history between two organisms, known as cospeciation (or codivergence) [14–17]. As previously mentioned, cospeciation is expected to occur especially between obligate biotrophic pathogens and their host plants, leading to a high ration of pathogen species to host species. However, it has been proven that convincing cases of cospeciation are indeed rare as cophylogenetic methods often overestimate the occurrence of cospeciation [18]. Recently, host shifts (also often called ‘host jump’) has been recognized as an important evolutionary driver of obligate plant pathogens to shift or to expand their host ranges, e.g. for oomycetes [19–21], powdery mildews [22–24], rusts [25], and smuts [12].

The downy mildews, the largest group of obligate parasites in oomycetes, are an especially attractive subject for macroevolutionary investigations in obligate biotrophs, because they represent a high number of species (more than 600 described ones), have high host specificity and occur on a great variety of different hosts in Angiosperms. In particular, the genus *Bremia* seems useful for detailed studies as it is of manageable size (so far about 15 accepted species) and has high specificity on a wide host range more than 40 genera and 200 species of the largest flowering plant family, Asteraceae (Compositae).

Thus, the aim of this study was to reconstruct each the phylogeny of the downy mildew genus *Bremia* and their Asteraceae hosts. We also date all nodes using the novel approach of maximum clade ages for species-specific subgroups of Asteraceae-parasitic downy mildews. These results allow us to reconstruct how the downy mildew species have successfully specialized and diversified on their host plants.

## Materials and Methods

### Oomycete and Asteraceae samples

In total 195 specimens of Asteraceae plants showing downy mildew infection were sampled (S1 Table). Two genera, *Novotelnova* and *Protobremia*, which were previously classified under *Plasmopara* or *Bremia* but recently introduced as new genera [5, 26], were also included, as the two genera are phylogenetically basal to *Bremia* with similar morphology, and their hosts, *Scorzonera* and *Tragopogon*, belong to the subfamily Cichorioideae of Asteraceae. *Paraperonospora tanaceti* and *Barnadesia odorata* were selected as outgroup taxa, in consideration of previous phylogenetic studies for *Bremia* [26] and Asteraceae [27], respectively.

### DNA extraction, PCR amplification, and sequencing

In total, 5–20 mg of infected plant tissue from herbarium specimens were taken and ground in a mixer mill (MM2, Retsch, Germany), using three iron beads of 3 mm diameter per sample. Genomic DNA was extracted using the BioSprint 96 DNA Plant Kit (Qiagen, Germany) on a Kingfisher Flex (Thermo Scientific, Germany). For oomycetes, PCR amplification and sequencing for the D1/D2/D3 region of 28S, partial 18S, ITS1 rDNA, and the partial *cox2* mtDNA follow Choi et al. [28], and those for *cox*1 mtDNA were identical to that of Robideau et al. [29] using the primers Oom-CoxI-lev-up and Oom-CoxI-lev-lo. For the RXLR11 gene, primers RxLR11-F (ATGCGCTACAAGCTTATCG) and RxLR11-R (TTACAAATCGAGCGTGTTT) designed by Joan Wong and Richard Michelmore (unpublished) were used for amplification. For Asteraceae PCR amplification was done using the plant-specific primer ITS1-P [30] and ITS4 [31] for ITS rDNA, and using the primers 3F-KIM (CGTACAGTACTTTTGTGTTTACGAG) and 1R-KIM (ACCCAGTCCATCTGGAAATCTTGGTTC) designed by the CBOL Plant Working Group (unpublished, http://www.barcoding.si.edu/plantworkinggroup.html) for the matK chloroplast region. Amplicons were sequenced at the Biodiversity and Climate Research Centre (BiK-F) laboratory using primers identical with those used for amplifications.

### Phylogenetic analysis

Sequences were edited using the DNAStar software package (DNAStar, Inc., Madison, Wis., USA), version 5.05. An alignment of each locus was performed using MAFFT 7 [32] version 6.0, employing the Q-INS-i algorithm [33]. To construct the plant and fungal phylogenies, we used the data derived from the concatenated sequences after checking that individual loci did not result in strongly supported conflicting topologies. To assess the relative stability of trees to methods of analysis, we used four different tree construction methods: Maximum Likelihood (ML), Maximum Parsimony (MP), Minimum Evolution (ME), and Bayesian (MCMC) methods. For ML analyses, 500 rounds of random addition of sequences as well as 500 fast bootstrap replicates were performed using RAxML 7.0.3 [34] as implemented in raxmlGUI 1.3 [35] using the GTRCAT variant. MP analysis was performed using 1000 replicates of heuristic search with random addition of sequences and subsequent TBR branch swapping in PAUP 4.0b10 [36]. All sites were treated as unordered and unweighted, with gaps treated as missing data. The reproducibility of the internal branches from the resulting trees was tested by bootstrap analysis using 1000 replications. ME analysis was done using MEGA 5.0 [37], with the default settings of the program, except for using the Tamura-Nei model. MCMC analysis was performed using the MrBayes Version 3.2 [38]. The general time reversible model with gamma-distributed substitution rates was employed. Four incrementally heated simultaneous Markov chains were run for 10M generations, with a tree saved every 1000th generation. The first 1K trees generated via this method were ignored. MrBayes was used to compute a 50% majority rule consensus of the remaining trees to obtain estimates for the posterior probabilities of groups. To test the reproducibility of results, the analysis was repeated four times, starting with random trees and default parameter values.

### Isolate selection for cophylogenetic analyses

**Fig 1:**
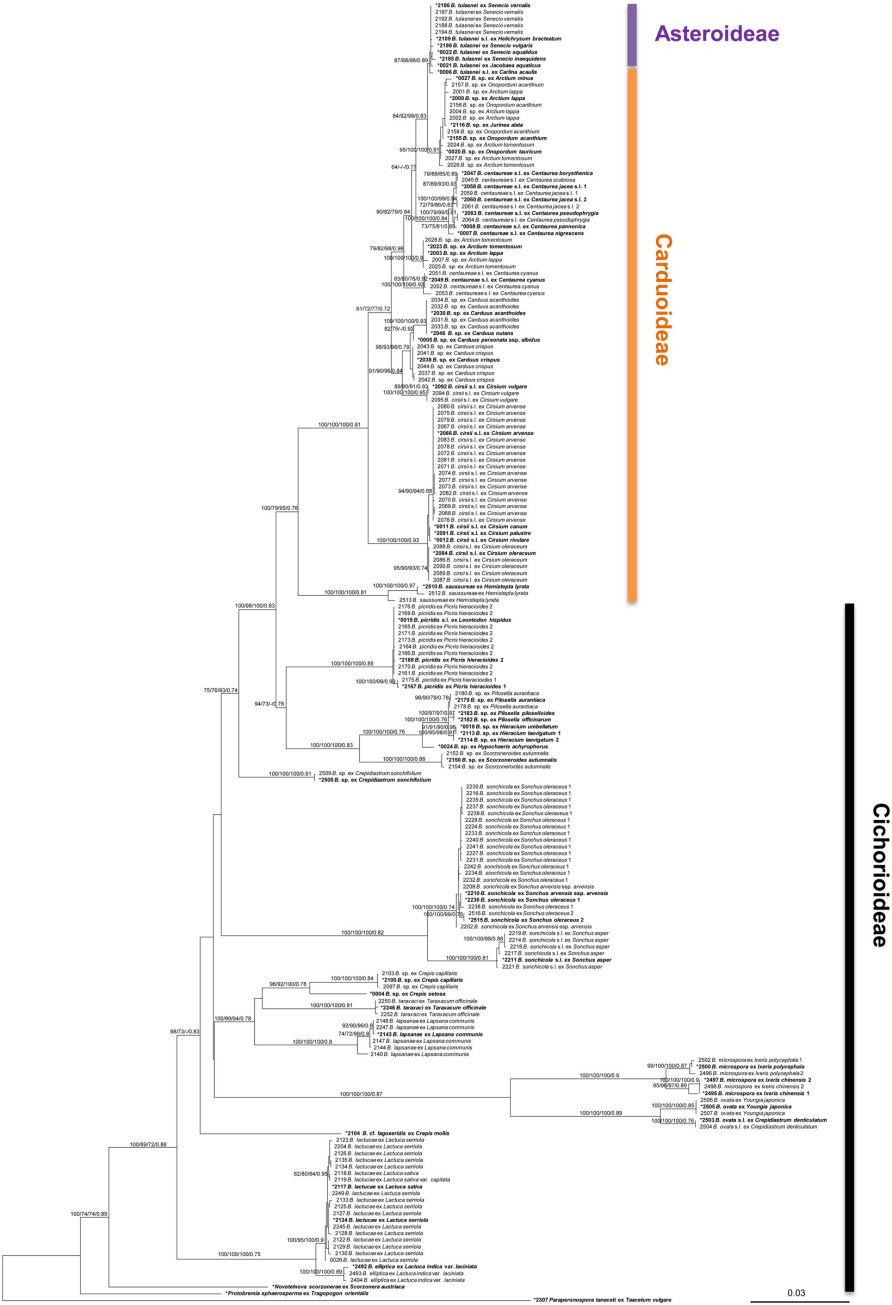
Maximum likelihood tree for *Bremia*, inferred from the combined sequences of *cox*2, *cox*1, the spacer region between *cox*2 and *cox*1 genes, 18S, 28S, ITS1, and *RxLR11* loci. ML, MP, ME bootstraps, and MCMC posterior probability more than 70% are shown above or below the branch. Asterisk (*) marks the samples selected for cophylogenetic analyses.

**Fig 2:**
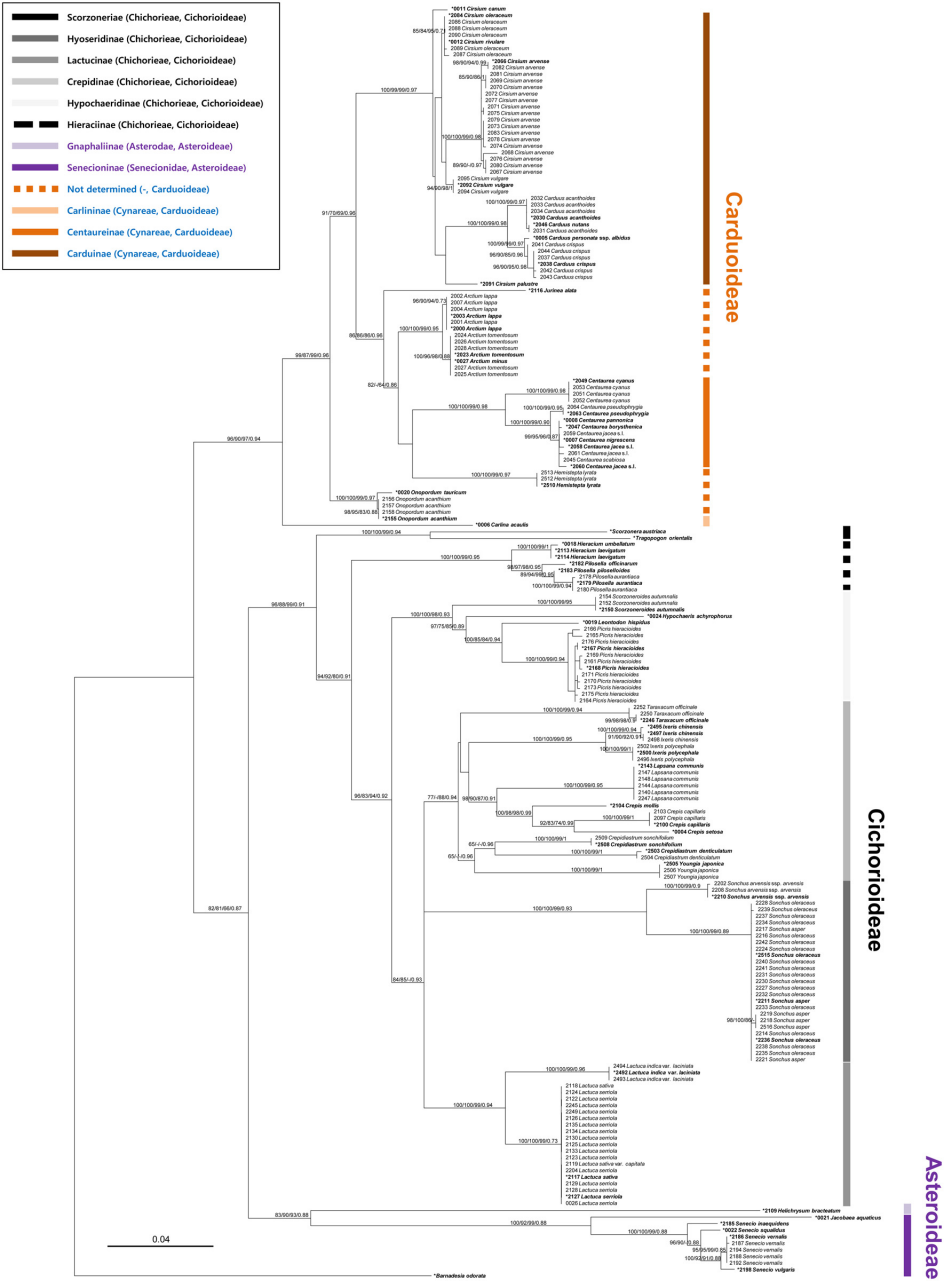
Maximum likelihood tree for Asteraceae, inferred from the combined sequences of matK and ITS loci. **ML, MP, ME bootstraps, and MCMC posterior probability more than 70% are shown above or below the branch**. Asterisk (*) marks the samples selected for cophylogenetic analyses.

For cryptic phylogenetic species of *Bremia* which were found in the present study but were not described previously, we used the criterion of phylogenetic congruence between different gene phylogenies and the distinctiveness of the grouping for considering a group of isolates as an independent evolutionary species of *Bremia*. Because of the large size of the host and parasite phylogenies (Figs 1 and 2), cophylogenetic analyses were performed on pruned topologies that included one representative downy mildew species per host species. For this, not only species already described, but also phylogenetic groups of parasites or host isolates were considered an independent evolutionary lineage, when they were 1) consistently found by at least three of the four reconstruction methods (ML, MP, ME, MCMC) in the concatenated phylogeny and 2) strongly supported as monophyletic with the values of ML bootstraps/ME bootstraps/MP bootstraps/ MCMC posterior probabilities at least equal to 70%. Based on this criterion, two specimens for *Centaurea jacea*, *Hieracium laevigatum*, *Ixeris chinensis*, *Picris hieracioides*, and *Sonchus oleraceus* and their pathogens were included in the pruned analyses.

### Preparation of the sub-datasets of cophylogenetic analysis

Using the 64 sequences selected, the phylogenetic analyses outlined above were performed again for downy mildews and Asteraceae. From the four trees inferred by ML, ME, MP, and MCMC analyses, a 75% consensus tree was constructed for each of downy mildews and Asteraceae. Pruning did not change tree topology, and had no effect on the analyses other than reducing computation time. We applied tree reconciliation methods to compare a range of trees representing alternative hypotheses of host and parasite relationships and identify those elements of the reconstructions that are shared across combinations of trees, Icong [39], TreeFitter 1.3.2 [40], Tarzan v0.9 [41], CoRe-PA 0.5.1 [42], Jane 4 [43], as well as a distance-based method, ParaFit [44]. Among them, Icong, TreeFitter, and Tarzan do not accept polytomies in topologies, and require trees with the same number of leaves, which allow neither a parasite (generalist) infecting more than two host plants nor a host plant infected by more than two parasites. On the other hand, CoRe-PA, Jane, and ParaFit programs do not require fully resolved phylogenetic trees and allow for handling parasites with multiple hosts, as it is the case in the present study. To overcome this problem, we built two different topologies for each of the plants and downy mildews; (A) phylogenetic trees with polytomies that account for both parasites associated with multiple hosts and host associated with multiple parasites, and (B) fully resolved trees with leaves linking several fungal or host species were duplicated as necessary. For the second dataset (B), the unresolved nodes with polytomy at the 75% consensus level were fully resolved when supported by significant statistical values (ML, ME, MP bootstraps and MCMC posterior probabilities at least equal to 70%) at least two of the four phylogenetic reconstruction methods in each of the two phylogenies.

### Cophylogenetic analysis on the basis of parasite and host trees

For the two datasets (A, B), we compared their phylogenetic trees by using two comparison methods, topology and distance. Topology-based methods use topologies to assess the fit between host and parasite phylogenies. The Icong index assesses the topological congruence of two trees through their maximum agreement subtree. Different from Icong, TreeFitter, Tarzan, CoRe-PA, Jane programs attempt to reconstruct the shared evolutionary history between hosts and their parasites minimizing the number of hypothesized historical events needed to explain the topological differences between hosts and pathogens, assigning mostly four types of potential events: codivergence (Co), duplication (Du), sorting or extinction of the parasite lineage (So), and host shift (HS). A disadvantage of these methods is that the number of cophylogenetic events is sensitive to selection of different phylogenetic hypothesis for evaluating cospeciation and event costs schemes. Taking these uncertainties into account, we applied six different event costs schemes for all cophylogenetic analyses (see Table 1); each default cost combination of Tarzan, TreeFitter, CoRe-PA, and Jane, and in addition two cost combinations estimated by CoRe-PA, for each of polytomy tree with generalists and hosts infected with multiple parasites (A) and binary tree with individual links (B), and an equal event cost.

**Table 1:** Results of the cophylogenetic analyses under various cost settings. The tests were performed using consensus trees inferred from the combined sequence data of *Bremia* and Asteraceae.

		**Tarzan**												
**Data set**	**Model**	**Event** ^**a**^ **costs**					**Events found**						**Compatibility** ^**b**^	
		Co	Du	HS	So	FD	Co	Du	HS	So	FD	Cost		
**B**	Optimized by Core-PA	7	11	21	3		n.a.	n.a.	n.a.	n.a.				
	Core-pa default	0	2	3	1	1	25	0	39	5	1	129	Yes	
	Jane4 default	1	1	2	1		n.a.	n.a.	n.a.	n.a.				
	Treefitter default	0	0	2	1		28	0	37	8	2	84	Yes	
	Tarzan default	-1	1	3	2		29	0	35	13	1	105	Yes	
	Equal weights	1	1	1	1	1	n.a.	n.a.	n.a.	n.a.				
		**TreeFitter 1.3**												
**Data set**	**Model**	**Event costs**					**Events found**						**p-value**	
		Co	Du	HS	So	FD	Co	Du	HS	So	FD	Cost		
**B**	Optimized by Core-PA	7	11	21	3		31^c^	10^c^	22^d^	51^c^		942	0.0197	
	Core-pa default	0	2	3	1		27–29^c^	0–1	33–36^d^	14–21^c^		122	0.0022	
	Jane4 default	1	1	2	1		17–23^c^	0	40–46^d^	0–6		109	0.0001	
	Treefitter default	0	0	2	1		23–28^c^	0–5	30–40^d^	6–26		86	0.0001	
	Tarzan default	-1	1	3	2		n.a.	n.a.	n.a.	n.a.				
	Equal weights	1	1	1	1		0–17	0	46–63	0		63	1	
		**JANE4**												
**Data set**	**Model**	**Event costs**					**Events found**						**p-value**	
		Co	Du	HS	So	FD	Co	Du	HS	So	FD	Cost		
**B**	Optimized by Core-PA	7	11	21	3	3	27	18	8	79	10	822	0.001	
	Core-pa default	0	2	3	1	1	25	0	28	32	10	126	0.001	
	Jane4 default	1	1	2	1	1	15	0	38	12	10	113	0.001	
	Treefitter default	0	0	2	1	1	22	3	28	24	10	90	0.001	
	Tarzan default	-1	1	3	2	2	23	0	30	23	10	133	0.001	
	Equal weights	1	1	1	1	1	0	0	53	9	10	72	0.001	
**A**	Optimized by Core-PA	14	11	262	2	2	27	36	0	194	0	1162	0.001	
	Core-pa default	0	2	3	1	1	27	0	36	18	0	126	0.001	
	Jane4 default	1	1	2	1	1	17	0	46	0	0	109	0.001	
	Treefitter default	0	0	2	1	1	24	2	37	15	0	89	0.001	
	Tarzan default	-1	1	3	2	2	23	0	40	9	0	115	0.001	
	Equal weights	1	1	1	1	1	0	0	63	0	0	63	1	
		**Core-Pa**												
**Data set**	**Model**	**Event costs**					**Events found**						**Robustness** ^**e**^	**Chronological valid**
		Co	Du	HS	So	FD	Co	Du	HS	So	FD	Cost		
**B**	Optimized by Core-PA	7	11	21	3		27	17	9	64		757	0.067/0.170/0.001	True
	Core-pa default	0	2	3	1		25	2	26	13		95	0.348/0.128/0.001	False
	Jane4 default	1	1	2	1		18	1	34	3		90	0.212/0.202/0.001	True
	Treefitter default	0	0	2	1		23	5	25	17		67	0.135/0.252/0.001	False
	Tarzan default	-1	1	3	2		23	1	29	10		85	0.262/0.331/0.001	False
	Equal weights	1	1	1	1		n.a.	n.a.	n.a.	n.a.				
**A**	Optimized by Core-PA	14	11	262	2		27	36	0	194		1162	0.011/0.165/0.001	True
	Core-pa default	0	2	3	1		29	0	34	20		122	0.602/0.668/0.001	False
	Jane4 default	1	1	2	1		20	0	43	3		109	0.517/0.292/0.001	False
	Treefitter default	0	0	2	1		27	4	32	22		86	0.599/0.526/0.001	False
	Tarzan default	-1	1	3	2		27	0	36	14		109	0.588/0.512/0.001	False
	Equal weights	1	1	1	1		n.a.	n.a.	n.a.	n.a.				

A: phylogenetic trees with polytomies that account for both parasites associated with multiple hosts and host associated with multiple parasites, B: fully resolved trees with leaves linking several fungal or host species.

n.a.: not available as the program do not support for the cospeciation event cost greater than zero.

^b^: Timing compatibilities between switches.

^c^: The number of events significantly exceeds that for randomized trees (*P* <0.01).

^d^: The number of events is significantly less than that for randomized trees (*P* <0.01).

^e^: the number indicates of cospeciations occurred by the 1000 random reconstructions (random trees for host / parasite / randomized host—parasite associations on the original trees).

TreeFitter uses a method based on generalized parsimony to assess the fit between the host and parasite phylogenies by adequately mixing the four basic types of events. The overall cost and occurrence of each event type was tested by 10,000 permutations. In the present study, co-phylogeny scenarios were reconstructed by using CoRe-PA software with pre-defined cost values and automatically calculated cost values for two sub-datasets, A and B (see Table 1). For significance evaluation, three randomization testings were performed with 1000 instances by creating random trees for host and parasite, or with randomized host—parasite associations on the original trees, respectively. The parastie tree was randomized under the beta = -1 model. In addition to the four types of coevolutionary events, Jane uses a fifth type named “failure to diverge (FD)”, referring to the instances when a host speciation is not followed by parasite speciation, which remains as the same species on the newly produced host species. In the present study, 500 generations and a population size of 50 were used as parameters of the genetic algorithm. Statistical tests were performed using 999 randomizations with random parasite trees.

Distance-based methods focus on the fit between host and parasite distances and do not test for the presence of any coevolutionary events. In this study, patristic distances (summed branch lengths along a phylogenetic tree) were calculated in CopyCat 2.02 [45] for each host and parasite phylogeny. The global fit between trees is computed and tested by randomizing individual host-parasite associations. ParaFit was used to test whether a particular host-parasite link contributed to this global fit. Tests of significance were performed using 999 permutations.

### Sequence divergence comparison and maximum clade age estimation

Comparison of evolutionary rates of host and parasite using divergence timings of the two phylogenies using fossil calibration points would be the ideal to determine whether hosts and their parasites have cospeciated simultaneously or need to be attributed to host-shift driven speciation. However, similar to other biotrophic pathogens, this approach is not applicable due to the scarcity of the fossil record available for Oomycetes. Instead, we used two different approaches. First, we compared their corresponding divergence rates using uncorrected distance matrices of each organism calculated in MEGA 5. The slope of the line is the relative divergence rate in sequences of host and parasite. If sequence divergence in hosts and parasites is correlated, then fitting the slope of line to a plot of parasite divergence should be similar to the host divergence regardless of the three subfamilies of host plants infected.

Second, we assigned maximum clade ages to groups that have a defined ancestral host range, making use of the fact that a pathogen clade originally infecting a certain group of hosts can be assumed not to be older than the divergence of the last common ancestor of that group. Estimation of the divergence times and evolutionary rates of host plants and downy mildews was performed with BEAST v1.7.4 [46]. Method and parameters for Asteraceae follow Tremetsberger et al. [27], using the oldest fossil ages of *Cichorium intybus* (min. 22 mya, max. 28.4 mya), *Sonchus oleraceus* (min. 5.4 mya), and *Scorzonera hispanica* (min. 3.4 mya) type pollens. Additionally, we assigned a node age of 47.5 million years for the Cichorioideae—Carduoideae split, representing the first reliable point for calibration of the split between Barnadesioideae and the rest of Asteraceae [47, 48]. As unfortunately there is no fossil record available to allow calibration of downy mildews, the maximum age of *Bremia* was inferred as 25.3 million years, the node age of the core group of Cichorieae to which all Cichorioideae plants that *Bremia* infects belong [27]. The resulting phylogenetic trees were summarized in TreeAnnotator version 1.7.4 [49] (using a 10% burn-in) and visualized using FigTree (http://beast.bio.ed.ac.uk).

## Results

### Downy mildew phylogeny

Based on a concatenated alignment of mitochondrial (*cox*2, *cox*1, the spacer region between *cox*2 and *cox*1 genes) and nuclear (partial 18S, D1/D2/D3 region of 28S, ITS1, *RxLR11*) loci, the infrageneric relationships of *Bremia*, including *Protobremia* and *Novotelnova*, were investigated using ML, MP, ME, and MCMC analyses. Trees based on single, non-concatenated loci showed no strong conflicting support with the multi-gene analyses based on the concatenated alignments. The mitochondrial and nuclear trees were largely congruent excluding a different position of a clade containing *Bremia ovata* and *B*. *microspora*. In the tree using mitochondrial loci only, this clade was placed basal to all other *Bremia* species investigated (including *Novotelnova* and *Protobremia*). But in the tree based on nuclear loci the *B*. *lactucae*/*B*. *elliptica* clade was placed basal to the remaining species of *Bremia*, with *Novotelnova* and *Protobremia* being placed basal to all *Bremia* species. However, the basal position in the tree based on the mitochondrial loci received only weak support (85% in ME, but less than 70% in other analyses).

In the multi-gene phylogeny (Fig 1), the distinctiveness of the lineages parasitic to specific host genera or species received strong to maximum supports in all phylogenetic analyses conducted, resolving the uncertainties found in previous studies due to the relatively small number of taxa and genes analyzed [50, 51]. Especially, the resolution for *Bremia* isolates parasitic on Asteroideae and Carduoideae was much improved. In addition, the phylogenetic analyses revealed that *Bremia* exhibits pronounced cryptic diversity with several distinct lineages, which mostly correspond to a particular host species.

### Asteraceae phylogeny

The phylogenetic analysis of the Asteraceae produced similar topologies for ITS rDNA and matK-based trees, but the ITS region showed a better discrimination at the species level. The reconstructions of the concatenated alignment of the two regions were not significantly different from the single locus topologies in ML, MP, ME, and MCMC analyses, although in the matK tree, grouping and branching of the genera belonging to Crepidinae were slightly different from ITS tree, however with low support values. As no highly supported conflicting topologies were inferred with the different phylogenetic methods used, only a ML tree of the concatenated alignments is presented (Fig 2), with the addition of the support values of the MP, ME, and MCMC analyses. All phylogenetic analyses resulted in three major groups, representing three subfamilies of Asteraceae, Asteroideae, Cichorioideae, and Carduoideae, in line with previous studies [52, 53]. The former two subfamilies further formed a group, although support was only moderate. In general, the phylogenetic clades inferred fit well with previous taxonomic divisions of Asteraceae, especially at the rank of subtribes of Cichorioideae [27, 54]. Among four genera of Carduoideae, *Jurinea*, *Arctium*, *Hemistepta*, and *Onopordum*, which were previously placed into neither Carduinae nor Centaureinae, the former three genera were found to be associated with the subtribe Centaureinae in the present study, but the *Onopordum* occupied an independent lineage, basal to both subtribes. At the genus level, there is an inconsistency between the present phylogenetic results and the current classification, with the genus *Cirsium* being paraphyletic; *Cirsium palustre* as sister taxon to the genus *Carduus* clade and *Cirsium canum* as sister to the combined clades of *Cirsium* and *Carduus*. Both markers used in the present study showed a good resolution mostly at the species level, but failed to differentiate between a few closely related species; *Lactuca sativa* vs. *L*. *serriola*, *Sonchus oleraceus* vs. *S*. *asper*, *Carduus acanthoides* vs. *C*. *natans*, and four *Centaurea* species.

### Topology-based cophylogeny

**Fig 3:**
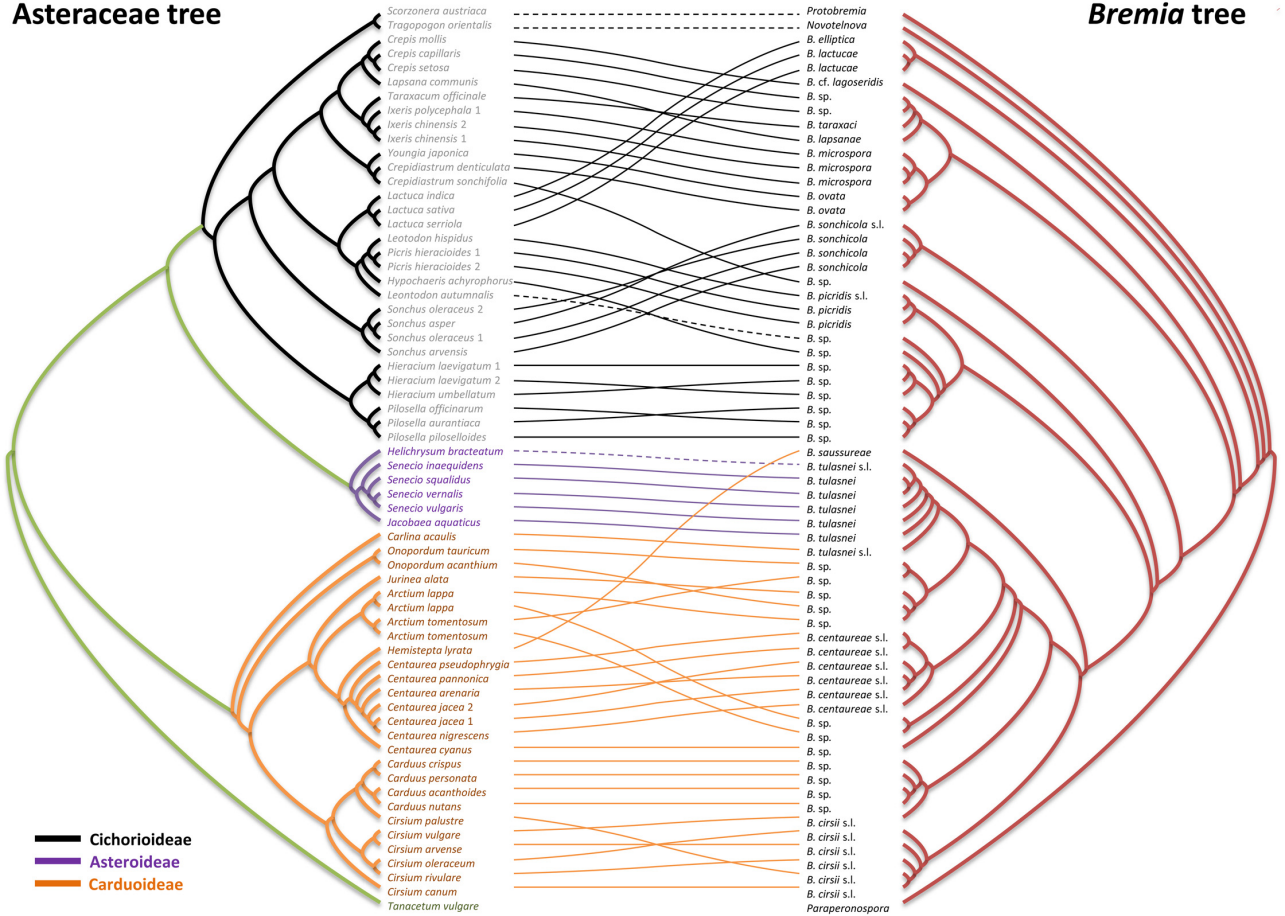
Tanglegram for the Asteraceae (left) and *Bremia* (right). Each tree is a consensus tree of ME, ML, MCMC, MP trees inferred from the combined sequences of matK and ITS genes for Asteraceae and *cox*2, *cox*1, the spacer region between *cox*2 and *cox*1 genes, 18S, 28S, ITS1, *RxLR11* loci for *Bremia*. Each leaf of *Bremia* is linked to its host. Bold lines indicate significant links between taxa (ParaFit, *P* < 0.05), and dashed lines indicate marginally significant links between taxa (ParaFit, *P* > 0.05). Tanglegram was produced in TreeMap 3b (https://sites.google.com/site/cophylogeny/).

All topology-based methods, as implemented in the Icong, Tarzan, TreeFitter, CoRe-PA, and Jane, were employed to analyze signatures of congruence of the phylogenetic trees inferred from the combined datasets of *Bremia* and Asteraceae. For simplicity we only show 75% consensus trees derived from ML, MP, ME, and MCMC trees, and their associations are shown as disentangled using TreeMap 3b (Fig 3). As the estimated numbers of each type of event depend on the cost assignments, event costs schemes of the six different combinations that were technically possible for all cophylogenetic analyses were applied (Table 1).

Icong found a maximum agreement subtree with 15 leaves for *Bremia* and Asteraceae phylogenies. The congruence between the two trees is significantly higher than that of random trees with the same leaf number (Icong index = 1. 2788, p-value = 0. 006126). Tarzan showed that there is a compatible structure in this host-parasite association for all three default cost assignments of Tarzan, TreeFitter, and CoRe-PA, available for this program. The numbers of codivergence and host shift events were always higher than duplication and sorting, regardless of different cost settings, signalling that these events are dominating the evolutionary history of the host-parasite system. TreeFitter revealed that there is a congruent structure in this host-parasite association, using all available cost assignments. The fit between the host and parasite phylogenies, tested by permutation, showed that the overall cost of all events is significantly lower than expected by chance alone (P < 0.01), demonstrating correspondence between the host and parasite trees, due to signals for cospeciation, as codivergence occurred significantly more frequently than in the randomized trees. For all cost structures excluding the equal assignment, host shift occurred less frequently than in randomized trees.

**Fig 4:**
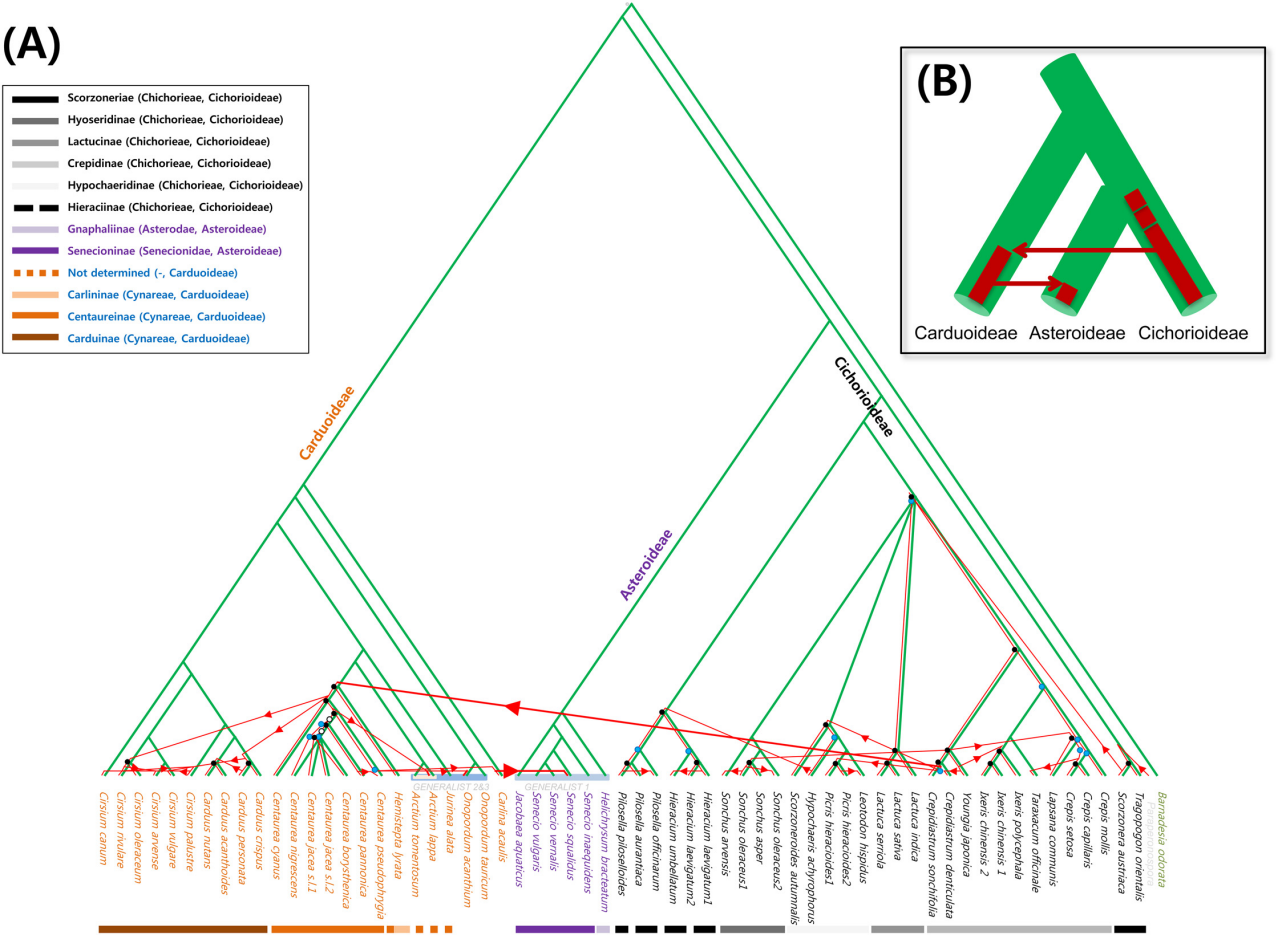
A potentially optimal reconciled tree between Asteraceae and *Bremia* inferred from CoRe-PA (A) and the simplified tree (B). One of several best-fit scenarios proposed by the program is shown, which includes 25 codivergence, 26 host-switches, 2 duplication, and 13 sorting events. Green and red lines represent the host and parasite, respectively. Four evolutionary events are denoted: codivergences (black circle), duplication (white circle), sorting events (blue circle), and host-switches (red line with arrowhead). Dashed red lines indicate distant host jumps across different subfamilies of Asteraceae.

CoRe-PA revealed a good compatibility between downy mildew and plant phylogenies. However, the numbers of four events inferred by CoRe-PA are divergent depending on the cost model chosen. The pre-defined cost values and those approximated automatically by the program for the optimal reconstruction where the cost vector fits best to the reconstructed event frequencies were vastly divergent (Table 1). When using the optimal inferred cost values for the dataset (A), reconciliation resulted in a maximum of 27 co-speciation events, which is a bit higher than for the four pre-defined cost models (18–24). However, the best-fit scenarios inferred much higher number of duplication (17) and sorting events (64), as well as a smaller number of host shifts (9) than the standard pre-defined models. A similar result was obtained when using the dataset (B). A possible co-phylogenetic scenario using the default cost combination of CoRe-PA is shown in Fig 4. To test if the observed amount of 25 co-speciations were significantly more than expected by chance, randomization tests were performed. A significance test with 1000 randomized host-parasite associations resulted in 99.9% of the solutions having fewer than 25 co-speciations and none having more and thus confirming a significant degree of congruence between parasite and hosts trees. Also in randomized host trees and randomized pathogen trees, the majority of the trees revealed less co-speciation events, with 87.2% and 65.2%, respectively. Similar results were obtained when other pre-defined cost values were used, for both datasets (A, B). However, at the non-default parameters the randomized tree tests did not confirm co-speciation above statistical noise. Interestingly, all best-fit scenarios with different standard cost schemes for the two datasets (A, B), excluding one case that used the automatically optimized cost for dataset B, postulated two distant host shifts of downy mildews to distantly related host plants, marked with a red dotted line in Fig 4. In these scenarios, long after the radiation of downy mildews on the Cichorioideae a host shift from Cichorioideae onto Carduoideae was observed, which corresponds to the observation that the basalmost clades of the *Protobremia-Novotelnova-Bremia* are parasitic to hosts in the Cichorioideae. The second shift from Carduoideae to Asteroideae occurred more recently, and apparently has not yet resulted in an adaptation.

In Jane 4, the reconciliation result indicated a strong topological congruence between *Bremia* and Asteraceae trees. The genetic algorithm using 500 generations and a population size of 50 found similar numbers of events as CoRe-PA (Table 1). The resulting potentially optimal reconciled trees also suggested that there were at least two independent host shift events, initially from Cichorioideae to Carduoideae and subsequently to Asteroideae. For a fifth type named “failure to diverge”, Jane 4 inferred ten such events for dataset A, but none for dataset B. The congruence between the phylogenies was highly significant in all randomization tests, even when using random parasite trees.

### Distance-based cophylogenetic analyses

ParaFit analysis was performed by calculating patristic distances from the ML tree of the combined data sets. The global test using ParaFit confirmed the results obtained by topology-based methods and inferred a significant correlation between parasite and host genealogies (ParaFitGlobal = 0.00156, P = 0.0001). The test of individual links (S2 Table) indicated that 60 links (out of 64) contribute to the global fit between the two data sets at significant level of 5%. These results show that only four associations, *Protobremia* parasitic on *Tragopogon*, *Novotelnova* on *Scorzonera*, and two *Bremia* species on *Helichrysum* and *Scorzoneroides*, do not contribute to the overall fit between parasites and hosts. Also when using uncorrected distances calculated from the combined data sets, the global test of cospeciation revealed a global association between hosts and parasites (ParaFitGlobal = 0.0022, P = 0.0001). This is suggestive of a high degree of cospeciation between downy mildew and Asteraceae, irrespective of the calculation method applied.

### Comparison of sequence divergences

**Fig 5:**
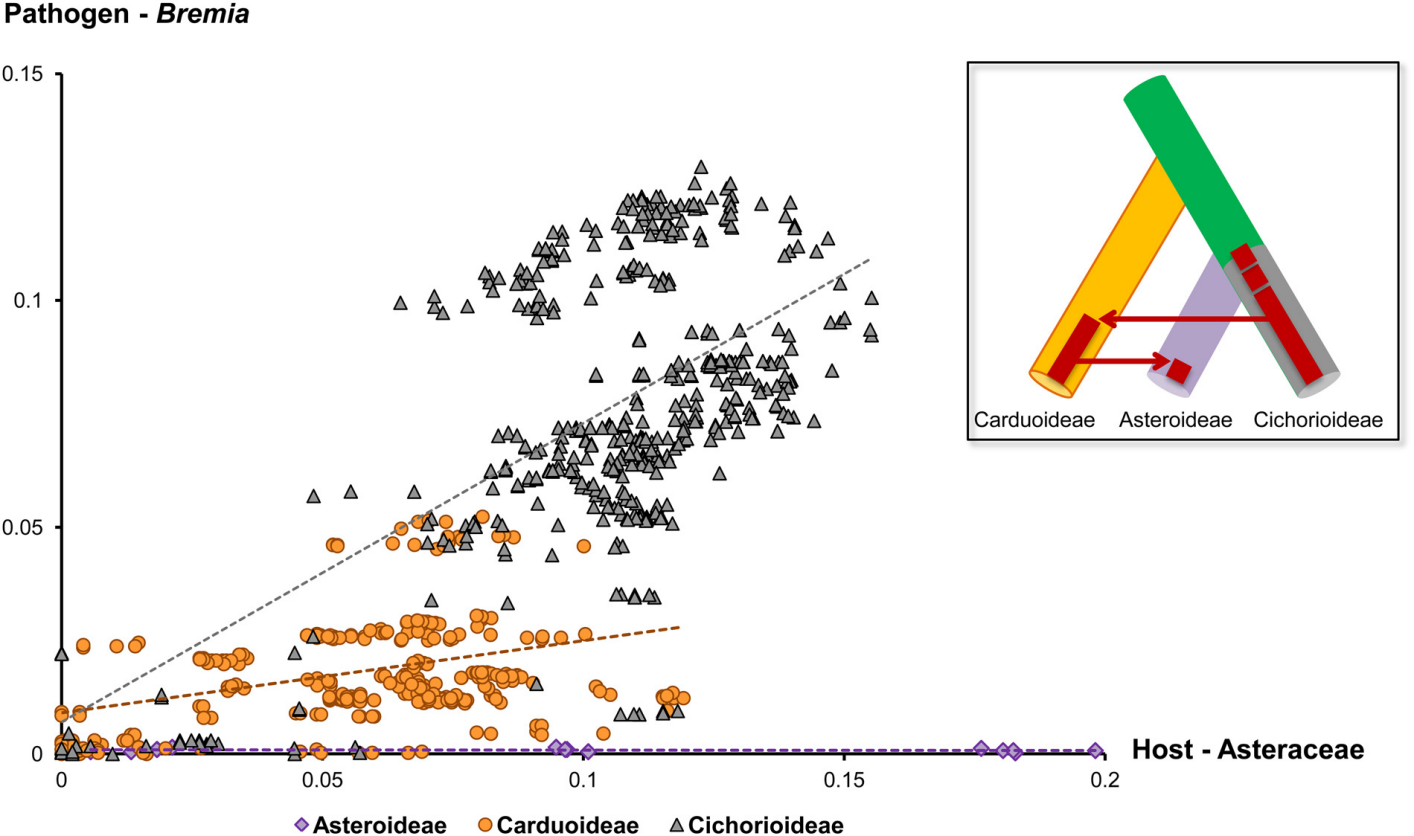
Bivariate plot of corresponding sequence divergences of pathogens and hosts at the level of species of Asteraceae. The inlay illustrates the evolution of *Bremia* by host jumps as inferred from cophylogenetic analyses, genetic divergence, and maximum clade ages.

To investigate in more detail, if the two wide host-shifts inferred by cophylogenetic analyses (from Cichorioideae to Carduoideae and further to Asteroideae), long after the initial radiation of downy mildews on the source subfamily, we compared sequence divergences among the downy mildew isolates parasitic on the three subfamilies of Asteraceae. In a bivariate plot of corresponding sequence divergences of downy mildew and Asteraceae (Fig 5), the three groups of downy mildews showed highly different divergence rates in relation to the three host subfamilies, thus supporting the results inferred from cophylogenetic analyses. Downy mildews on Cichorioideae showed the highest divergence rate, downy mildews on Carduoideae revealed a modest rate, and the downy mildews on Asteroideae exhibited the lowest rate, being not significantly different from zero. Also the sequence divergences among downy mildew isolates from widely divergent host genera (e.g. between *Helichrysum* and other plants) of Asteroideae are very low (less than 0.1%) or even identical. In addition, a comparison at the level of host genus, to avoid a possibly biased result towards closely related parasites on closely related host plants was carried out, the plot (S1 Fig) showed a similar scattering and a similar slope of the regression with results from the species level, indicating that the pattern is not strongly influenced by phylogenetic distance. Drawing these results together, downy mildews radiated on Cichorioideae and after some time lag jumped to Carduoideae to radiate again in this group. Then, after another time lag, they jumped to the Asteroideae where they radiated again without producing significant genetic divergence among lineages, so far.

### Correlation of coalescence times by molecular clock

**Fig 6:**
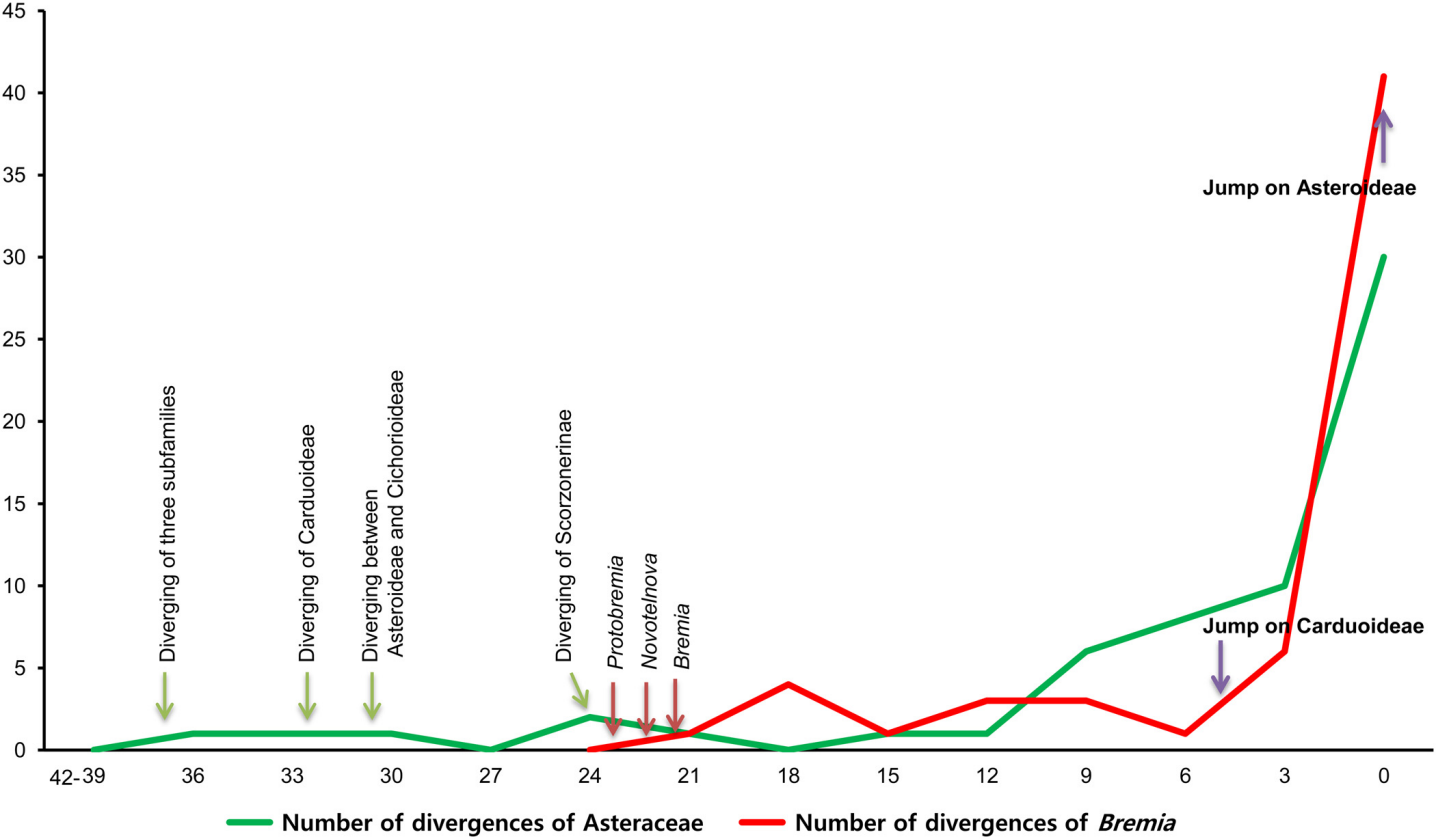
Number of divergence events counted in every 3 million years in each molecular clock tree of Asteraceae and *Bremia*.

Hypotheses of cospeciation imply that speciation events in hosts and parasites should be contemporaneous. While fossil records exist for Asteraceae, there is no fossil record available for downy mildews, an indirect approach based on maximum clade ages had to be used (i.e. the age of a pathogen subgroup specialized on a certain host group can for parsimony reasons be assumed not be older than the corresponding host species group). We counted and compared the lineage divergence events for every 3 million year period in the mapped molecular clock trees of downy mildews and Asteraceae. If downy mildews and host plants have indeed cospeciated, then it is expected that the number of lineage divergences in their mapped dated trees show a similar changing pattern over time. For Asteraceae, the BEAST program calculated a rate of 11.75 x 10^−9^ substitutions per site per year with good statistical precision, which is slightly faster than the rate reported previously for Asteraceae (Tremetsberger et al. [27]; 10.7–10.8 x 10^−9^ substitutions x site^-1^ x year^-1^). For downy mildews, this implies that the ancestors of the genus *Bremia* are at most between 22 and 27 myr old (Fig 6), although this should be interpreted with caution, given that the inference is based on maximum clade ages inferred from the host trees. The true age might thus be significantly younger. Diversification of host plants has increased fast between 12–9 myr, and then accelerated 9 myr ago, while *Bremia* diversified more during the last 6 myr. Especially during the past 3 myr, the diversification slope of *Bremia* is steeper than for its host plants, indicating that the divergence speed of downy mildews was faster than that of its host plants. This provides additional support for the inference that there has not been synchronous cospeciation.

## Discussion

Over the past 10 years, an increasing amount of genome data for well-known pathogens has been advancing the understanding of the biology and genetics of oomycetes and other pathogens. But there are still insufficient genomes available for concluding on evolutionary questions with respect to species diversification. Many studies have addressed the phylogenetic relationships of pathogens, and especially in downy mildews, many groups can be considered to be well-studied in this respect. *Hyaloperonospora arabidopsidis*, a specific parasite of *Arabidopsis thaliana*, has become a pathogen model, so the understanding of the relationship has been gained about the interactions between oomycetes and their host plants at the molecular level [9]. Highly host-specific downy mildews are also attractive models for co-evolutionary studies. As obligate plant pathogens have been in intimate association with the hosts during their diversification, cospeciation was generally believed as a driving evolutionary event, resulting in a parallel speciation pattern between parasite and host phylogenies. Although there are a few direct comparisons using cophylogenetic methods for obligate plant pathogens and host plants [11, 12], taxonomic sampling and phylogenies of pathogens and plants were usually insufficient, in order to estimate an accurate evolutionary relationship between them. In the highly specialized and diverse downy mildews, a rigorous evaluation of cophylogenetic relationships had not yet been conducted previously, although some studies commented on the fact that, while there are some clades with restricted host spectra, a multitude of host jumps is needed to explain the overall phylogenetic patterns [3–5, 21]. The present analyses revealed that the dominant pattern in the evolutionary history of downy mildews has not been strict cospeciation, but that diversity has been shaped by host shifts and subsequent radiation and speciation. First, all analyses based on the pre-defined costs in CoRe-PA program inferred that the parasite and host trees are chronologically inconsistent. Second, most of the strong congruent links between downy mildews and host plants are extremely biased towards terminal branches, while being weak in internal nodes. Third, the potentially optimal trees reconciled using CoRe-PA and Jane, as well as comparisons based on sequence divergence and molecular clock data also supported more recent speciation of downy mildews, long after radiation and diversification of the host plants.

Especially the radiation after a major host shift is a phenomenon that has not been previously considered for obligate pathogens and host plants, but is suggested by the data obtained for the genus *Bremia*, as the divergence times and genetic differentiation between parasites and hosts are vastly different. This agrees with two major host jumps associated with radiation followed by specialization and speciation, rather than true cospeciation with contemporary divergence of hosts and pathogens. This is especially apparent in the *Bremia* lineages that evolved after a host jump from Carduoideae to Asteroideae, which are parasitizing deeply divergent lineages in the old genus, *Senecio*, but are genetically barely differentiated. In the light of these findings the codivergence events found in present analyses are mostly based on radiation and subsequent speciation rather than true cospeciation, the widespread assumption that host and pathogen congruence is due to cospeciation as a major mode of pathogen diversification should be reconsidered and previous findings be re-evaluated using a maximum clade age approach, considering also genetic divergence patterns. Only including the temporal dimension and considering genetic divergence patterns direct evidence was obtained that downy mildews, which can probably stand exemplary for other plant-pathogen systems, have not co-diverged with their hosts. This highlights the importance of considering the temporal dimension in studies on plant-pathogen coevolution.

There is no unambiguous report of convincing cospeciation in obligate plant pathogens of either fungi or oomycetes [18]. In the present study it was observed that significant phylogenetic congruence between hosts and their symbionts can indeed be obtained after a series of host shifts to the closest relatives of the original host in a radiation process rather than cospeciation, providing evidence for a concept that has independently also been suggested by de Vienne et al. [18, 55]. Interestingly, the present result revealed two distant host shifts across three host subfamilies of Asteraceae, the largest flowering family, demonstrating that host jumps to unrelated plants as well as closely related ones can occur frequently. Following a few experimental studies that host jumps should occur less frequent with a decreasing relatedness of the hosts [56–58], which is in line with the assumption that pathogenicity effector targets will be more divergent in distantly than in closely related hosts, it is plausible that the more frequent host shifts to closely related hosts will mimic co-speciation. However, the distant host jumps to largely unrelated hosts are probably the most important for the diversification of plant pathogens and downy mildews as these shifts would lead to a potential radiation in a large group of new hosts that are naïve to the pathogen, having a basal defence that has been overcome in related species, but no effector triggered immunity, yet [59]. The two distant shifts of downy mildews across three subfamilies of Asteraceae occurred rather recently, and in Asteroideae, a coevolutive adaptation has apparently not yet led to speciation. With increasing specialization pressure exerted by the hosts through the onset of evolutionary arms races, host ranges will become smaller and limited to closely related hosts, eventually ending in specialization on a single host species, as observed in many species of *Peronospora* [60–62]. If this is achieved, co-speciation might also occur, but will most likely be restricted to terminal nodes. Previously, host shift even across different plant orders have been shown in a downy mildew genus *Pseudoperonospora* [21] and the white blister rust *Albugo* [20]. In *Pseudoperonospora*, the host shift was occurred recently, with the two lineages of *Pseudoperonospora cubensis* still being in the radiation phase, although some specialization can already be observed [63]. The genus *Hyaloperonospora* has radiated in the comparatively young family Brassicaceae and already acquired significant genetic divergence, although most species are still pathogens of the Brassicaceae family, while some host jumps from this family to other families can be observed [4, 64]. The deeply rooted genera *Plasmopara* and *Peronospora* have already undergone several radiations, so its original host has not yet been identified [3, 65]. This again highlights that rather than continuous coevolution, host jumps, radiation, and subsequent specialization shape the diversity of downy mildews. Also among obligate plant pathogenic fungi, there are several cases where divergence patterns are implausible without host shifts, e.g. for powdery mildew [22–24], rust [25], and smut [12]. Therefore, host shift is recognized as a common major evolutionary event of obligate plant pathogens of fungi and oomycetes to shift or to expand their host ranges. To date the processes leading to a host shift are not well understood. However, given that the obligate plant pathogenic fungi and oomycetes obtained the biotrophic parasitism independently in the course of their evolution, this commonality in their macroevolutionary histories supports that it might constitute a general driver in the evolution of biotrophic pathogens.

When two phylogenies have different diverging times or ages, the cophylogenetic methods will be prone to overestimate the occurrence of cospeciation, resulting in false conclusions regarding host-pathogen coevolution. Comparison of evolutionary rates using homologous genes or of divergence timings using fossil records would be necessary to determine whether a pairs of hosts and their parasites have cospeciated simultaneously and whether a codivergence event found by cophylogenetic software is a real cospeciation or host-shift speciation. In most cases [12, 14, 66], including the present study, however, there is no detailed fossil record available. Instead, in the present study, maximum clade ages according to host spectra of pathogen groups were assumed. Recently, there was an attempt to calibrate the origin of oomycetes [67], using two diatoms and one oomycete fossils, although the latter fossil is still questionable whether it belongs to oomycetes and is far away from the tips, as it is about 400 Myr old. Using different molecular clock models, they have estimated the origin of *Phytophthora* as 23–27 Myr. This age is slightly older than the divergence of three closely related genera, *Bremia*, *Novotelnova*, and *Protobremia* (21–24 Myr), which was calibrated by the maximum age estimation method in the present study, not unexpectedly with a lower degree of variation as the single oomycete fossil used by Matari and Blair [67] dates back to the Devonian. Thus, even although this method will most likely still overestimate pathogen clade ages, it is a conservative approach that could be an alternative to fossil calibration to determine whether pairs of hosts and their parasites have co-speciated simultaneously or not.

The present study provides evidence that the evolutionary patterns of the biotrophic oomycetes are much more complex and dynamic than previously thought. Rather than strict coevolution and co-speciation, the evolution of the downy mildews and probably also of other obligate plant pathogenic groups have been shaped by host jumping, radiation and specialization, before entering strict coevolution, which might mostly only carry on for a limited time until either the pathogen is removed from the host population or the pathogen escapes the evolutionary arms-race by a host jump.

## Supporting Information

S1 Fig Bivariate plot of corresponding sequence divergences of *Bremia* at the level of genera of Asteraceae. (PDF)

S1 Table Summary of herbarium specimens used in this study (XLSX)

S2 Table The result of ParaFit analyses conducted using patristic distances of the ML trees. Probabilities are based on 999 permutations. Significant p-values (in bold) suggest that the link under evaluation has a significant contribution to the global fit, indicating significant associations between hosts and parasites. Probabilities in red, orange, green, and black are significant at levels of 1, 2, 5%, and insignificant links even at 5%, respectively.
(XLSX)
