# Supplementary figures and images for: Host Jumps and Radiation, Not Co‐Divergence Drives Diversification of Obligate Pathogens. A Case Study in Downy Mildews and Asteraceae
Source: PLoS One. 2015 Jul 31;10(7):e0133655. doi: 10.1371/journal.pone.0133655 (PMC4521919; doi:10.1371/journal.pone.0133655)

Pathogen - *Bremia*

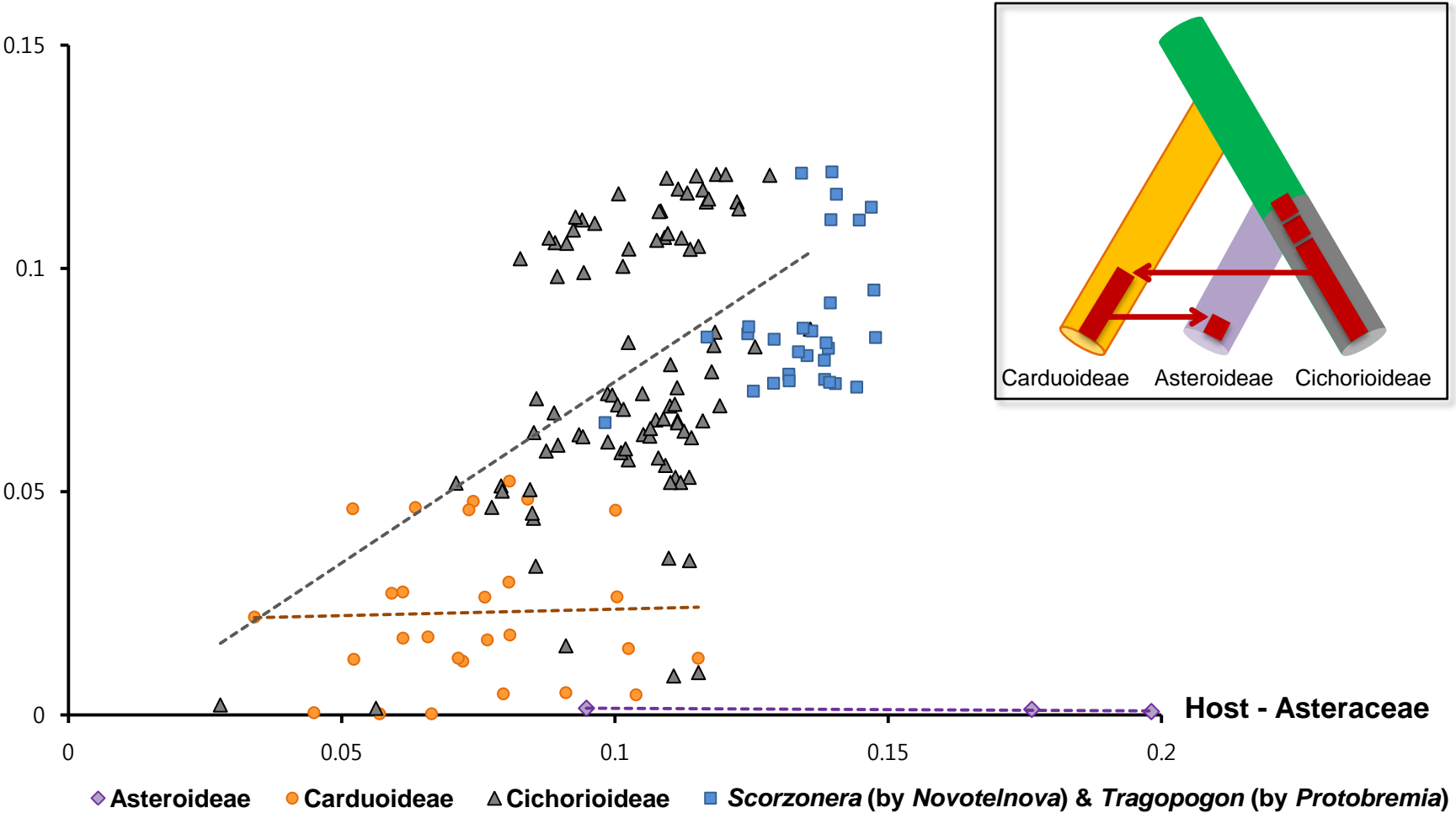

Supplement: S1 Fig — (PDF) [file pone.0133655.s001.pdf]
